# Diabetic Ketoacidosis in a Patient With Type 2 Diabetes Following Ophthalmic Steroid Use: A Rare Etiology

**DOI:** 10.7759/cureus.101283

**Published:** 2026-01-11

**Authors:** Raad Alwithenani, Nasser Al Shanbari, Abdullah Alshamrani, Waad M Malibarey, Yasser Alghamdi, Ammar Alzoriri, Bandar Alotaibi

**Affiliations:** 1 Department of Internal Medicine, King Abdulaziz Medical City, Ministry of National Guard Health Affairs, Jeddah, SAU; 2 College of Medicine, King Saud Bin Abdulaziz University for Health Sciences, King Abdullah International Medical Research Center, Jeddah, SAU; 3 Department of Internal Medicine, King Abdulaziz Medical City, Jeddah, SAU; 4 Department of Endocrinology and Metabolism, King Abdulaziz Medical City, Jeddah, SAU; 5 Department of Adult Diabetology, King Abdulaziz Medical City, Jeddah, SAU

**Keywords:** diabetic ketoacidosis, ophthalmic corticosteroids, steroid-induced hyperglycemia, topical steroid therapy, type 2 diabetes mellitus

## Abstract

The systemic adverse effects of corticosteroids are well established. However, steroid-induced diabetic ketoacidosis (DKA) has been rarely reported, and hyperglycemia resulting from topical ophthalmic corticosteroids remains insufficiently described, despite their widespread use and perceived safety.

We report the case of a 70-year-old female patient with longstanding type 2 diabetes mellitus who presented with DKA following the postoperative use of topical steroid eyedrops. The condition resolved within 24 hours with appropriate management, including isotonic fluid resuscitation, intravenous insulin infusion, and electrolyte replacement. Secondary causes of DKA were excluded through a thorough evaluation and multidisciplinary cooperation.

This case provides clinical evidence of the systemic metabolic effects of ophthalmic corticosteroids, particularly in patients with poor glycemic control. It highlights the importance of interdisciplinary communication and underscores the need for further studies to better define the risk of topical corticosteroids on glycemic control.

## Introduction

The hyperglycemic effect of corticosteroids has been reported in approximately 64-71% of patients who were initiated on systemic therapy [1,2]. These effects can result in deterioration of glycemic control in individuals with diabetes or precipitate new-onset diabetes in previously non-diabetic patients [3,4]. Corticosteroids induce hyperglycemia by increasing hepatic gluconeogenesis and glycogenolysis and by exacerbating insulin resistance through inhibiting the GLUT4 translocation at the adipose and muscle tissue [5,6].

Systemic corticosteroids are well known for their metabolic adverse effects; therefore, topical formulations are often considered safer alternatives [7]. However, studies have demonstrated that topical steroid administration may result in mild systemic absorption, leading to measurable increases in cortisol levels [8]. Additionally, transient elevation in blood glucose levels has been reported following the use of steroidal eye drops [9].

Although steroid-induced DKA is rare and hyperglycemic crises related to topical corticosteroids are poorly documented, these adverse effects should not be overlooked. We present a case of diabetic ketoacidosis (DKA) in a patient with type 2 diabetes following the recent initiation of an intensive regimen of ophthalmic corticosteroids.

## Case presentation

A 70-year-old woman with a known history of type 2 diabetes mellitus and multiple comorbidities, including ischemic heart disease, dyslipidemia, and knee osteoarthritis, presented with an acute onset of fatigue and reduced oral intake. Laboratory evaluation revealed severe hyperglycemia associated with high anion gap metabolic acidosis (Table 1). There were elevated serum osmolality and markedly increased serum beta-hydroxybutyrate (Table 1), consistent with DKA.

**Table 1:** Laboratory Findings

Laboratory Parameter	Laboratory Findings	Reference Range	Unit
Blood glucose	25 mmol/L	70–100	mg/dL (3.9–5.6 mmol/L)
Arterial pH	7.17	7.35–7.45	pH
Serum bicarbonate	11.7 mmol/L	22–28	mmol/L
Anion gap	21 mmol/L	7–13	mmol/L
Serum osmolality	311 mOsm/kg	285–295	mOsm/kg
β-hydroxybutyrate	9.67 mmol/L	<0.6	mmol/L

She was diagnosed with type 2 diabetes 23 years after a history of gestational diabetes during her last pregnancy. She was compliant with her treatment regimen, which included basal insulin (20 units daily), premeal insulin (15 units three times daily), metformin 1000 mg twice daily, and sitagliptin 100 mg once daily. She monitored her glucose levels using a continuous glucose monitoring system, with usual home readings ranging from 4 to 16 mmol/L. Her most recent hemoglobin A1C was 9.8% measured two months prior to presentation. She had no prior history of hyperglycemic crisis or diabetes-related renal or cerebrovascular complications. Her ischemic heart disease had been managed with percutaneous coronary intervention three years earlier, and she also had diabetic neuropathy controlled with analgesics.

Thirteen days prior to admission, the patient underwent uncomplicated glaucoma surgery and was discharged on prednisolone acetate 1% ophthalmic drops administered every two hours. Following initiation of this regimen, her home glucose readings progressively increased to values exceeding 20 mmol/L, despite adherence to her insulin therapy and reduced oral intake.

Alternative precipitating factors were systematically excluded; the patient demonstrated appropriate understanding and adherence to her insulin regimen, supported by a family member. There were no clinical or laboratory signs of infection, including a normal white blood cell count and negative inflammatory markers. Electrocardiography showed a normal sinus rhythm and cardiac enzymes were normal with no evidence of acute ischemia.

During her hospitalization, she received continuous intravenous fluid resuscitation and insulin infusion, with frequent adjustments guided by her volume status, electrolyte levels and metabolic parameters. Electrolyte abnormalities were corrected accordingly. Within 24 hours, her DKA resolved, evident by normalization of pH (7.36), bicarbonate (20.9 mmol/L), blood glucose (10 mmol/L) and serum ketone levels (2.32 mmol/L). She transitioned to subcutaneous insulin, tolerated oral intake, and was discharged with close ophthalmology follow-up for adjustment of her steroid eye drops.

## Discussion

DKA is one of the most serious acute metabolic complications of diabetes, alongside the hyperosmolar hyperglycemic state (HHS) [10]. DKA is characterized by a triad of uncontrolled hyperglycemia, metabolic acidosis and elevated circulating ketone levels, whereas HHS presents with severe hyperglycemia, hyperosmolality and dehydration without significant ketosis [10]. These metabolic derangements result from absolute or relative insulin deficiency, leading to increased secretion of counterregulatory hormones including glucagon, catecholamines, cortisol, and growth hormone [10]. DKA most commonly occurs in patients with autoimmune type 1 diabetes; however, it can also develop in individuals with type 2 diabetes, particularly in the setting of catabolic stress such as infection, trauma or surgery [10]. Approximately 68% of reported DKA cases occur in patients with type 1 diabetes, while about 32% occur in those with type 2 diabetes, although the true burden of DKA in the type 2 diabetes population remains incompletely defined [11]. 

Topical ophthalmic therapies, including corticosteroids, may lead to systemic adverse effects due to the absorption into the systemic circulation through the conjunctival capillaries, nasal mucosae, the oropharynx or via the gastrointestinal absorption after drainage through the nasolacrimal system [12]. Hyperglycemia is a recognized systemic effect once corticosteroids reach the circulation. In the United Kingdom, corticosteroid exposure has been associated with an odds ratio of 1.36 for developing new-onset diabetes [13]. Similarly, data from the New Jersey Medicaid program demonstrated an odds ratio of 2.23 for developing new-onset diabetes following corticosteroid use [5]. Furthermore, a large meta-analysis of 93 studies involving 6602 patients reported an odds ratio of 1.7 for new onset diabetes among individuals receiving systemic corticosteroids [14]. A retrospective study in an elderly population with a mean age of 74.9 years reported an odds ratio of 2.31 for corticosteroid-induced diabetes, with an incidence rate of 12% [15]. The dose and duration of corticosteroid therapy are considered the strongest predictors for the development of steroid-induced diabetes [16]. 

The mechanisms underlying corticosteroid-induced hyperglycemia are well established and include reduced insulin sensitivity leading to impaired glucose tolerance, increased glucagon secretion, enhanced basal hepatic glucose production, and reduced glucose uptake in adipose tissue [17]. While these metabolic effects may be compensated for by normal physiological mechanisms in healthy individuals, they can significantly exacerbate pre-existing insulin resistance in patients with diabetes, particularly in those with poor glycemic control [17].

Although corticosteroids are widely recognized to cause systemic adverse effects, including hyperglycemia, topically administered ophthalmic corticosteroids are generally perceived as safe and are rarely reported to cause hyperglycemia or hyperglycemic crises such as DKA [18]. Nevertheless, this report provides, to our knowledge, one of the very few documented cases demonstrating corticosteroid-induced DKA associated with ophthalmic steroid use in a patient with poorly controlled type 2 diabetes. In our patient, alternative precipitating factors for hyperglycemic crisis, including insulin noncompliance, infection, trauma, surgery-related stress, and acute cardiovascular events, were systematically excluded. However, she was at particularly high risk for developing a hyperglycemic crisis due to poor baseline glycemic control (HbA1c 9.8%), insulin dependence, advanced age, and the intensive dosing regimen of ophthalmic corticosteroids.

Consistent with our findings, a previously reported case described a 29-year-old patient without a prior history of diabetes who developed DKA after using prednisolone acetate 1% ophthalmic drops four times daily for one month following retinal detachment surgery [19]. Additionally, a published poster reported a 64-year-old male without known diabetes who developed significant hyperglycemia and elevated hemoglobin A1c levels after prolonged use of dexamethasone eye drops for chronic uveitis [20]. Collectively, these reports suggest that postoperative or prolonged use of ophthalmic corticosteroids may adversely affect glycemic control, particularly in susceptible individuals [17].

The cornerstone of DKA management includes prompt isotonic fluid resuscitation, intravenous insulin therapy with close glucose and potassium monitoring, and appropriate electrolyte replacement to maintain serum potassium levels between 4 and 5 mEq/L [20]. Frequent biochemical monitoring is essential to guide therapy and prevent complications related to overcorrection [20]. In accordance with the American Diabetes Association guidelines for the management of hyperglycemic crises, our patient achieved complete metabolic resolution within 24 hours without complications. Multidisciplinary collaboration was instrumental in identifying the precipitating factor and optimizing management to prevent recurrence [20].

This case highlights a rare and underrecognized etiology of DKA and underscores that DKA, although uncommon, can occur in patients with type 2 diabetes under specific circumstances. Importantly, it reinforces that ophthalmic corticosteroids are not metabolically benign, particularly in patients with poor glycemic control. Awareness of this potential complication is essential, and effective interdisciplinary communication is crucial for identifying precipitating factors and preventing severe metabolic decompensation.

Further studies, including additional case reports and observational analyses, are warranted to better characterize the risk and burden of hyperglycemia, new-onset diabetes, and hyperglycemic crises associated with topical ophthalmic corticosteroids. Such data may help inform preventive strategies and optimize glucose monitoring and management in high-risk populations.

## Conclusions

This case describes a rare presentation of DKA precipitated by postoperative ophthalmic corticosteroid use in an elderly patient with poorly controlled type 2 diabetes mellitus. The diagnosis was established after careful exclusion of other common precipitating factors for hyperglycemic crises. Early recognition and multidisciplinary management, involving close collaboration between endocrinology and ophthalmology, were essential for prompt resolution and prevention of recurrence.

This report underscores that ophthalmic corticosteroids are not metabolically benign, particularly in individuals with limited glycemic reserve. Clinicians should maintain a high index of suspicion for worsening glycemic control or hyperglycemic crises in high-risk patients receiving topical steroid therapy. Further studies are needed to better define the metabolic risks associated with ophthalmic corticosteroids and to guide appropriate monitoring and preventive strategies in vulnerable populations.
